# Multiplexing intensity and frequency sensations for artificial touch by modulating temporal features of electrical pulse trains

**DOI:** 10.3389/fnins.2024.1125597

**Published:** 2024-06-04

**Authors:** Kevin K. W. Ng, Alwin So, Jun Yi Fang, Ingvars Birznieks, Richard M. Vickery

**Affiliations:** ^1^Center for Social and Affective Neuroscience, Department of Biomedical and Clinical Sciences, Linköping University, Linköping, Sweden; ^2^School of Biomedical Sciences, UNSW Sydney, Sydney, NSW, Australia; ^3^Neuroscience Research Australia, Sydney, NSW, Australia; ^4^Bionics and Bio-robotics, Tyree Foundation Institute of Health Engineering, UNSW Sydney, Sydney, NSW, Australia

**Keywords:** tactile, electrical stimulation, burst, psychophysics, human, peripheral nerve, frequency discrimination, magnitude estimation

## Abstract

In neural prostheses, intensity modulation of a single channel (i.e., through a single stimulating electrode) has been achieved by increasing the magnitude or width of each stimulation pulse, which risks eliciting pain or paraesthesia; and by changing the stimulation rate, which leads to concurrent changes in perceived frequency. In this study, we sought to render a perception of tactile intensity and frequency independently, by means of temporal pulse train patterns of fixed magnitude, delivered non-invasively. Our psychophysical study exploits a previously discovered frequency coding mechanism, where the perceived frequency of stimulus pulses grouped into periodic bursts depends on the duration of the inter-burst interval, rather than the mean pulse rate or periodicity. When electrical stimulus pulses were organised into bursts, perceived intensity was influenced by the number of pulses within a burst, while perceived frequency was determined by the time between the end of one burst envelope and the start of the next. The perceived amplitude was modulated by 1.6× while perceived frequency was varied independently by 2× within the tested range (20–40 Hz). Thus, the sensation of intensity might be controlled independently from frequency through a single stimulation channel without having to vary the injected electrical current. This can form the basis for improving strategies in delivering more complex and natural sensations for prosthetic hand users.

## Introduction

We previously discovered a neural code for frequency perception in the human tactile system, which confers the advantage of encoding frequency in a manner independent of the number of spikes evoked in peripheral afferents (Birznieks and Vickery, 2017; Ng et al., 2018). When we grouped spikes into trains of periodic bursts, perceived frequency was best explained by the duration of the silent gap between bursts, rather than by the periodicity, mean spike rate or number of spikes within the burst (Birznieks and Vickery, 2017; Ng et al., 2020). The role of the number of spikes within a burst is not certain, but it is hypothesised that they encode other qualitative features of the stimulus such as intensity (Kaczmarek et al., 1992; Sharma et al., 2022c). One technology to generate such temporal spiking patterns in peripheral afferents is by very fast mechanical pulses with durations comparable to an action potential (Birznieks et al., 2019). For broader application in neural prosthetics (Vickery et al., 2020), we have verified experimentally that the same coding scheme can be implemented using electrocutaneous stimulation (Ng et al., 2020, 2021) and auditory click stimulation (Sharma et al., 2022a,2022b).

Firing patterns of action potentials grouped into temporal bursts have been posited to allow sensory neurons to encode multiple stimulus features on a fine temporal scale and also to enhance transmission robustness (Kepecs and Lisman, 2003; Mease et al., 2017). This form of multiplexed coding, where both overall spike count and precise spike timing carry information (Panzeri et al., 2010; Lankarany et al., 2019), enables greater information coding capacity than a simple rate code, as demonstrated in thalamic (Mease et al., 2017), auditory (Eyherabide et al., 2008) and visual (Reich et al., 2000) neurons. A form of multiplexing for frequency and intensity has been previously observed in the primary somatosensory cortex (S1) of primates by Harvey et al. (2013), where they found that information for vibrotactile stimuli is encoded at different time scales. Specifically, vibratory amplitude is represented by the coarse overall firing rate in a subpopulation of neurons, whereas frequency composition is encoded in the phase-locked temporal patterning of the neuronal response. Lankarany et al. (2019) propose another form of multiplexing in S1 for aperiodic stimuli, where the rate of asynchronous spiking encodes stimulus intensity, while the timing of synchronous spikes encodes abrupt changes in the intensity, including the occurrence of high contrast features such as edges.

The possibility of independently controlling perceived frequency and intensity using electrocutaneous burst stimuli as a form of sensory feedback has been previously proposed by Menia and Van Doren (1994). Their subjects performed pitch (frequency) matching of stimuli with different charges (proportional to pulse width multiplied by amplitude) and burst periodicity in one experiment, and loudness (intensity) matching with varying burst periods in others. They found that subjects’ pitch matches depended only on the burst period and were not affected by discriminable differences in charge. Moreover, subjects’ loudness matches were not affected by discriminable differences in burst periods of ±10 ms, and only depended slightly on burst periods over the range of 15.6 ms to 500 ms (corresponding to burst rates of 2.0–64.1 Hz). However, Menia and Van Doren only used stimuli with a fixed number of pulses within a burst, i.e., 10 pulses per burst.

Here, we test whether modulating the number of pulses within a burst might change intensity perception within the flutter range (20–40 Hz), by combining this strategy with our previously uncovered “burst gap” frequency coding scheme. The unprecedented advantage of this approach is that stimulus intensity could be rendered independently from frequency in a brain-machine interface, through one and the same stimulation channel, at a fixed stimulus strength.

## Methods

### Subjects

We conducted three separate experiments, where healthy human subjects with no history of altered tactile function were recruited. In Experiment 1, 14 subjects (5 females, ages 19–25) participated. For Experiment 2, we had 12 subjects (8 females, ages 20–25). Twelve subjects (4 females, ages 19–25) participated in Experiment 3, eight of which had also participated in Experiment 1. This study was approved by the UNSW Sydney Human Research Ethics Committee (approval number HC16245/210271). Prior to the start of experimentation, written informed consent was obtained from all subjects.

### Stimulation patterns

To test our hypothesis, we created four stimulus patterns that were expected to have identical perceived frequencies, as determined by the silent period between bursts regardless of the number of pulses within a burst (Birznieks and Vickery, 2017). These patterns (Doublet, Triplet, Quadruplet and Pentuplet) were bursts consisting of 2 to 5 pulses evenly spaced over a burst duration of 13.5 ms. Each burst was separated by an interval of 36.5 ms, and thus should all render a perceived frequency of ∼27 Hz. These values were chosen such that they were within the burst parameters tested in a previous study (Ng et al., 2020). Additionally, all burst patterns had the same periodicity, i.e., burst rate, of 20 bursts/s. The perceived frequency of these test stimuli was verified in Experiment 1.

In Experiment 2, subjects rated the intensity of some of the aforementioned stimulus patterns, including one consisting of regularly-spaced pulses (Singlet), which was designed to match the 36.5 ms burst gap and corresponding reciprocal perceived frequency of 27 Hz of the burst patterns. We have previously shown that each transcutaneous nerve stimulation pulse within these stimulus patterns reliably and consistently evokes neural activity in tactile afferents even at short inter-pulse intervals, and that these patterns all evoke the same perceived frequency (Ng et al., 2020; Sharma et al., 2022c).

**FIGURE 1:**
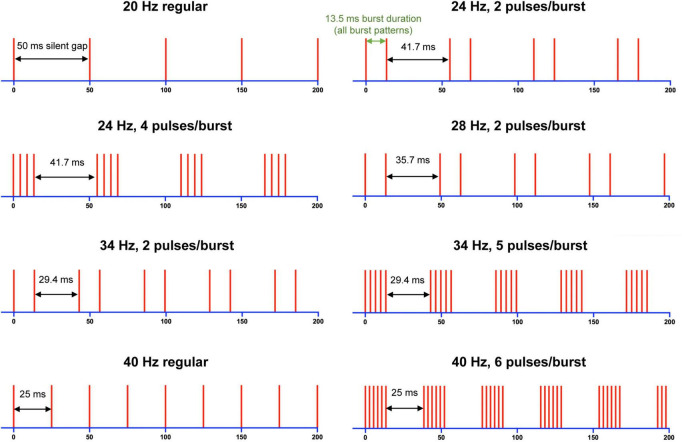
Schematic representation of stimulation patterns used in Experiment 3. Vertical lines represent an electrical pulse, and silent (burst) gap intervals are labelled for each stimulus train. Silent gaps in ms correspond to the frequency in Hz shown above each train. Note that a stimulus pattern, 20 Hz (6 pulses/burst) with 50 ms burst gap, was also tested but not shown here.

To also demonstrate that subjects can simultaneously, and independently, perceive both frequency and intensity, we conducted a further third experiment where subjects rated both parameters. Nine stimulus patterns were created, with either 1, 2, 4, 5, or 6 pulse(s) in the burst (Figure 1). As before, each burst had a duration of 13.5 ms, but the silent gap between bursts was varied (between 50 and 25 ms) to have corresponding reciprocal frequencies between 20 and 40 Hz.

### Experimental set-up

Stimulus patterns were generated using a CED Power1401 Mk II data acquisition system via Spike2 (Cambridge Electronic Design, Cambridge, UK) and MATLAB (Mathworks, Natick, MA, USA) software. This triggered a DS5 constant current stimulator (Digitimer, Welwyn Garden City, UK) to output charge-balanced, biphasic electrical pulses. The electrical pulses were delivered to one digital nerve of the index finger, on the subject’s dominant hand using Kendall 200 series foam electrodes (Covidien, Mansfield, MA, USA). One electrode was placed on the proximal phalanx and another on the distal interphalangeal joint.

**FIGURE 2:**
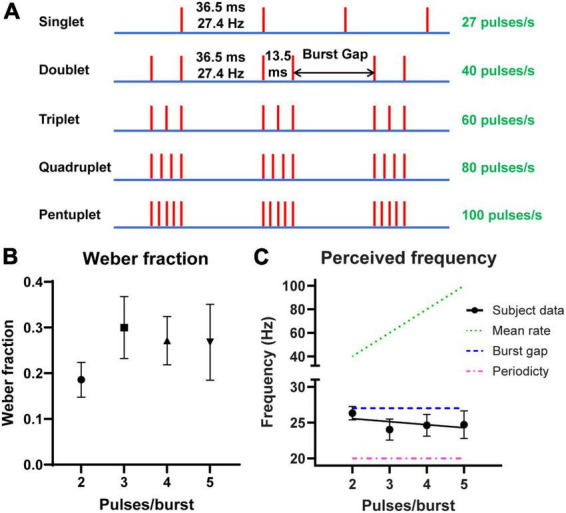
Stimulus patterns with similar perceived frequency despite varying number of pulses. **(A)** Schematic representation of stimulation patterns used in Experiments 1 and 2. Each vertical line denotes an electrical pulse. Numbers on the right represent the overall pulse rate for each stimulus train. **(B)** The mean Weber fractions (*n* = 14 subjects) for the tested burst stimuli in Experiment 1. This expresses the just noticeable difference (JND) in frequency change as a percentage of the test stimulus. **(C)** The mean perceived frequency (circles, *n* = 14 subjects) of the tested burst stimuli in Experiment 1. Point of subjective equality (PSE) was best predicted by the burst gap code corresponding to the reciprocal of the silent interval, i.e., ∼27 Hz, shown as a dashed line. The dotted line indicates frequency if it was predicted by the mean pulse rate of the stimuli, and the dash-dotted line if it was predicted by the periodicity, i.e., burst rate of 20 Hz. A regression line has been fitted to the subject data. Error bars indicate 95% CI.

The stimulation current applied to each subject was optimised such that the stimulus was clearly perceptible and distinguishable, confirmed by subjects being able to perform a practice task. The 40 Hz (6 pulses/burst) pattern (Figure 1) was used for this in Experiments 1 and 3, and the Quadruplet pattern (Figure 2A) was used in Experiment 2. The maximum current used was 10 mA. The pulse waveform was charge balanced as a 0.1 ms cathodic pulse, followed by 1 ms at 10% current in the reverse polarity to allow for charge to be recovered from the electrode (Hofmann et al., 2011).

### Psychophysical tasks

#### Two-alternative forced choice

In Experiment 1, the perceived frequency of the stimulus patterns was assessed using a two-alternative forced choice (2AFC) method. Subjects were presented with pairs of stimulus patterns and asked to press a button according to which they felt had a higher frequency or rate of “tapping”. Each trial consisted of a test stimulus, which was one of the burst patterns (with either 2, 3, 4, or 5 pulses per burst, Figure 2A), and a comparison stimulus, with regularly-spaced pulses at either 18, 21, 25, 29, 33, or 37 Hz. These were presented for 1 s each and in a random order, with 0.5 s in between. There were approximately 120 pairs for each test condition, with each comparison stimulus presented 20 times. A 5-min break was provided between each of the four condition blocks tested. Subjects were given practice stimuli and received feedback to ensure their understanding of the task.

#### Magnitude estimation

In Experiments 2 and 3, the perceived intensity of all stimulus patterns was assessed using the psychophysical paradigm of magnitude estimation (Stevens, 1956). In each trial, subjects were presented with a pair of stimuli consisting of the standard stimulus first, followed by one of the test patterns second. The stimuli were each presented for 1 s, separated by a 500 ms interval. Subjects were instructed to consider the standard stimulus as having an intensity of 100 arbitrary units and to verbally report the relative perceived intensity of the test stimulus. For example, if they considered the second stimulus in the pair as two times as intense, they would report a value of “200”, and if half, they would report “50”. Subjects were again given practice stimuli similar to test conditions. For Experiment 2, the stimulus pattern with periodic bursts of 2 pulses (Doublet) was chosen as the standard and was compared against one of four patterns – Singlet, Doublet (itself), Triplet and Quadruplet. Each test pattern was presented 20 times.

In Experiment 3, the standard stimulus was the 28 Hz (2 pulses/burst) stimulus (Figure 1), and this was compared against one of the 9 stimulus patterns (including itself). Additionally, subjects also rated the magnitude of frequency, in addition to intensity and in a similar manner. Subjects completed a block with 10 presentations of each comparison, rating them either for frequency or intensity relative to the standard. The order of whether subjects rated intensity or frequency magnitude first was randomised.

### Data analysis

To determine each subject’s perceived frequency of the test stimulus in Experiment 1, the number of times that subjects judged that test stimulus as having a higher frequency than that of the comparison stimuli was recorded. This proportion was logit transformed and a regression line was fitted to the data. The point of subjective equality (PSE) was taken from the x-intercept of the fitted regression line, representing the frequency where the subject perceived the test stimulus as being equally higher or lower than that comparison frequency. This PSE value would be their perceived frequency of the test stimulus. Furthermore, each subjects’ Weber fraction, which calculates the percentage change in the stimulus that can be reliably detected, was calculated using the halfway point between the frequencies that give 25 and 75% response probabilities for each test stimulus. Both calculations were performed in Excel (Microsoft, Redmond, WA, USA). A one-way repeated measures ANOVA (with Geisser-Greenhouse correction) compared the Weber fractions between the four different stimulus patterns, including *post hoc* Tukey’s multiple comparisons testing between pairings, with Prism software (Graphpad Software, San Diego, CA, USA).

For Experiments 2 and 3, subjects’ magnitude ratings were recorded manually in Excel and an average was calculated for each stimulus pattern. In Experiment 2, a regression line was fitted between the ratio of the mean pulse rate (1.0 = 40 pulses/s) and the ratio of intensity ratings averaged across subjects using Prism.

In Experiment 3, we first plotted the average frequency rating of all subjects for each of the 9 stimulus patterns against that of intensity. On this, regression lines were fitted based on arbitrary values chosen to show the ability to control intensity in either direction (increasing and decreasing trends) with a change in perceived frequency. Regression lines were subsequently fitted to determine if average frequency ratings correlated to changes in the burst gap predicted frequency, as well as whether there was a relationship between pulses per burst and average intensity ratings in Prism.

## Results

### Experiment 1: verifying perceived frequency of burst patterns

In the first experiment, we used a 2AFC procedure to measure perceived frequency of stimuli with a fixed burst gap duration, but varying number of pulses per burst (Figure 2A). Subjects’ PSE values ranged from 21 to 28 Hz for the different patterns and had an overall mean of 24.9 ( ± 2.1 SD) Hz. The Weber fraction for each test pattern is shown in Figure 2B. The overall Weber fraction between all subjects and conditions was 0.25 ± 0.11 (mean ± SD). Differences were found between Weber fractions of the stimulus patterns (F_2.168, 28.19_ = 6.431, *p* = 0.0042), e.g., when the doublet was compared against the triplet (*p* = 0.0029) and quadruplet (*p* = 0.0122), but not the pentuplet (*p* = 0.1437). Nonetheless, the data match the 0.2–0.3 range reported in the literature (Bull et al., 1985; Li et al., 2018; Graczyk et al., 2022).

The mean PSE for each test pattern is shown in Figure 2C. The slope of the fitted regression line was not significantly different from zero (−0.42, 95% CI −1.06 to 0.21, *p* = 0.1873) with a y-intercept of 26.41 (95% CI 24.07–28.74). This suggests that there was no trend between perceived frequency and pulses per burst (R^2^ = 0.03). While the periodicity (burst rate) of the four patterns was also constant at 20 Hz, the reciprocal of the silent burst gap, i.e., 27 Hz, remains a better predictor of perceived frequency.

### Experiment 2: rating perceived intensity of burst patterns

**FIGURE 3:**
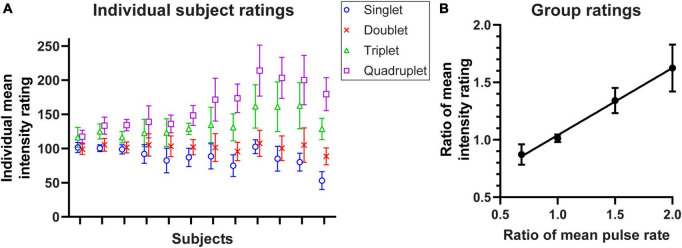
Perceived intensity of stimulus patterns shown in Figure 2A and tested in Experiment 2. **(A)** Mean ratings (*n* = 20 repeats) for the four tested stimulation patterns in individual subjects (*n* = 12), sorted by subjects’ reported intensity range; error bars depict SD. **(B)** The ratio of the intensity ratings relative to 100 averaged across 12 subjects, plotted against the ratio relative to the mean pulse rate of the Doublet standard stimulus (1.0 = 40 pulses/s); error bars denote 95% CI. A regression line has been fitted to the data.

Subjects were tested with pairs of 1 s stimulus trains where the first was always the standard stimulus (Doublet, Figure 2A), and then asked to rate the relative intensity of the second stimulus. The results are summarised for 12 subjects in Figure 3A, which show a consistent intensity of around 100 when the Doublet was compared against itself (crosses). As the number of pulses in the burst increased from 1 to 4 (3× increase in mean rate), the perceived intensity increased by 1.82× (median; quartiles 1.47× and 2.34×).

In the group mean data, perceived intensity increased linearly with the mean number of pulses per second (Figure 3B). The slope of the fitted regression line was 0.58 (95% CI 0.47–0.69; R^2^ = 0.70; *p* < 0.0001) which indicates that although a doubling in the number of pulses did not double the perceived intensity, the increase was significant and clearly perceptible.

### Experiment 3: simultaneous magnitude ratings of frequency and intensity

**FIGURE 4:**
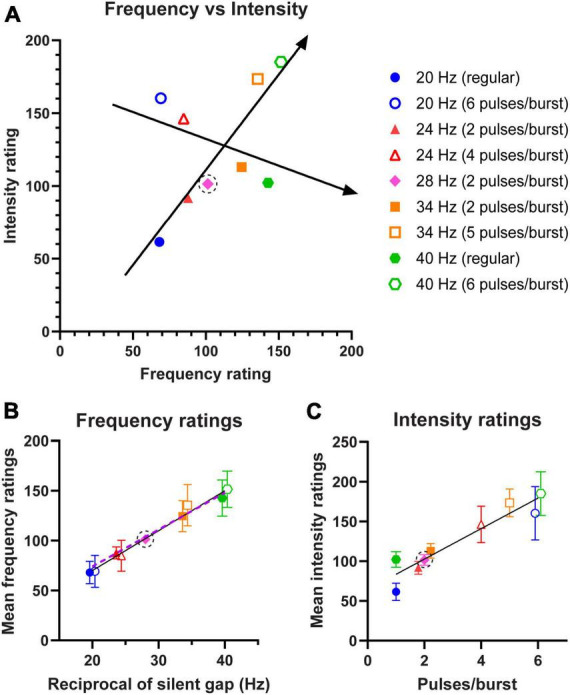
Average magnitude ratings of frequency and intensity for stimulus patterns shown in Figure 1 and tested in Experiment 3. All stimuli were compared against the standard of 28 Hz (2 pulses/burst), which is marked with a dotted circle. Unfilled shapes have equivalent predicted frequencies to filled shapes, but a greater number of pulses per burst. **(A)** Mean frequency ratings plotted against intensity ratings (*n* = 12 subjects) for the 9 stimulation patterns. A regression line is fitted to the sequence predicted to show increasing frequency and intensity (20 Hz regular, 28 Hz 2 pulses, 34 Hz 5 pulses, 40 Hz 6 pulses), and to the sequence predicted to show a decreasing trend of intensity magnitude ratings despite increasing frequency (20 Hz 6 pulses, 24 Hz 4 pulses, 24 Hz 2 pulses, 40 Hz regular). **(B)** Average frequency ratings plotted against expected frequency corresponding to the reciprocal of the silent gap, with regression line fitted. The dashed line depicts predicted frequency ratings assuming the relationship was linear. **(C)** Average intensity ratings plotted against number of pulses per burst, with regression line fitted. Data points jittered horizontally in panels **(B,C)** to minimise overlap. Error bars represent 95% CI.

In Experiment 3, we tested whether varying the number of pulses per burst would be able to cause subjects to report a higher frequency stimulus as less intense than a stimulus with a lower frequency, and vice versa. The results for subjects’ magnitude ratings of their perceived frequency and intensity of the stimulus patterns are summarised in Figure 4A. Subjects consistently responded with values around “100” when the standard was compared against itself in both perceived frequency and intensity ratings. The results also show that increasing the frequency corresponding to reciprocal of the silent gap would result in a report of higher perceived frequency (slope = 1.31, R^2^ = 0.80, *p* < 0.0001); whilst decreasing pulses per burst would result in subjects reporting a lower intensity rating despite frequency increasing (slope = −0.37, R^2^ = 0.13, *p* = 0.0134).

In Figure 4B, the slope of the fitted regression line for average frequency ratings was 4.00 (95% CI 3.41–4.59). In Figure 4C the slope of the regression line was 19.17 (95% CI 16.06–22.28) for the average intensity ratings. Both these demonstrate a significant linear positive relationship between the frequency (R^2^ = 0.63, *p* < 0.0001) or intensity ratings (R^2^ = 0.58, *p* < 0.0001), and the frequency corresponding to the silent gap or the pulses per burst, respectively. Furthermore, it is evident in Figure 4B that stimuli with the same predicted frequencies were rated as having similar frequencies and that doubling the frequency from 20 to 40 Hz caused a twofold increase in the frequency ratings. The frequency rating was remarkably linear, with 20 Hz (6 pulses) being rated as 69 relative to the standard stimulus of 28 Hz, giving an equivalent frequency of 19.3 Hz, and similarly the 40 Hz (regular) was rated 142 relative to the standard which translates to a frequency of 39.8 Hz. Intensity ratings shown in Figure 4C display a marked increase between the lowest and highest number of pulses, with the increase of 59% due to a doubling from 2 to 4 pulses per burst matching the equivalent increase seen in Experiment 2. This demonstrates that within the zone of 20 and 40 Hz we are able to arbitrarily vary frequency and also modulate intensity up and down without changing individual stimulus pulse properties.

## Discussion

A previous study by Szeto (1985) proposed a logarithmic relationship between pulse width and pulse rate, whereby the pulse width of an electrotactile pulse train must decrease if its pulse rate increases, to maintain a constant intensity, but variations in the pulse rate would result in concurrent changes to perceived frequency. Our results clearly demonstrate that it is possible to control intensity perception by varying the number of pulses in a burst, while maintaining the same stimulation current (and pulse width) and so fixing the population of activated afferents. Combined with our previous studies showing the ability to control frequency by the gap between bursts even if overall pulse rate was varied (Birznieks and Vickery, 2017; Ng et al., 2021), this might represent a method of multiplexing intensity and frequency information in a peripheral nerve stimulation pattern. Therefore, what we have established here undoubtedly allows intensity to be varied independently from frequency, and the reverse to a degree. In order to control frequency independently from intensity, one would still need to take into account the relationship between the stimulus pulse train pattern, mean pulse rate and intensity, which is not necessarily linear and may depend on perceived frequency (Ng et al., 2022). For example, in our data the 40 Hz (regular) stimulus is felt as more intense than 20 Hz (regular); and to balance the intensity at these different frequencies, the lower frequency would need to have more spikes per burst than the high frequency.

The results are comparable with Muniak et al. (2007) and Bensmaia (2008), which showed intensity varied with firing rate with a positive slope relationship of less than 1 in different afferent types; and Kaczmarek et al. (1992), which showed that increasing the number of pulses in a burst from 1 to 6 almost doubled the average magnitude in subjects. Several other studies have also suggested the use of varying number of pulses within bursts as a method of providing a wide range of sensations without eliciting pain (Collins, 1970; Saunders, 1974). For example, Sachs et al. (1980) measured a slope of 1.8 using electrical stimulation with changing the number of pulses per burst, but they had used cross-modality matching with the magnitude of auditory tones, which may explain the difference with our results. Similarly, Sharma et al. (2022c) demonstrated that temporal structuring of a fixed number of electrical pulses over a second into periodic bursts of varying pulses altered perceived intensity as a function of burst pulse count, however stimuli in that study were not controlled for changes in frequency perception.

Along with the principal debate of how afferent activity translates into a perception of stimulus intensity, previous studies have noticed substantial inter-subject variability (Knibestöl and Vallbo, 1980). Knibestöl and Vallbo (1980) demonstrated that power functions fitted to the relationship between indentation amplitude and perceived intensity are highly individualistic – the exponents ranged from 0.36 to 2.09 in their study and were not related to differences in neural activity. This is consistent with our observations (Figure 3), and we further speculate that subjects who showed the smallest effects on intensity may have interpreted pulse number within the burst as affecting some other qualitative feature of the stimulus such as sharpness, roughness or fuzziness, in addition to tactile intensity (Steenbergen et al., 2012; Tezuka et al., 2016; Kaczmarek et al., 2017).

The present findings were obtained using non-invasive electrocutaneous stimulation, which could be implemented with targeted muscle/sensory reinnervation approaches (Kuiken et al., 2007; Hebert et al., 2014). Nevertheless, we expect that the results would also be applicable when using electrodes implanted directly in the nerve to deliver sensory feedback (Tan et al., 2014; Graczyk et al., 2016). A particular benefit provided by our method is that intensity sensation could be varied in a single channel to increase dynamic range without increasing stimulation current, thereby reducing potential adverse effects on tissues in the vicinity of implanted electrodes (Cogan et al., 2016; Günter et al., 2019) and the likelihood of discomfort from recruitment of smaller diameter, higher threshold nociceptive afferents (Hallin and Torebjörk, 1973; Kaczmarek et al., 1991).

Implementing the principles established here within single channel stimulation with complex activity patterns across multiple electrodes offers the promise of providing multiple channels of parallel information as used in biomimetic approaches to restoring touch (Saal and Bensmaia, 2015; Okorokova et al., 2018). During surface exploration and object manipulation, vibrations of various frequencies arise depending on the dynamics of the movement (Delhaye et al., 2012; Shao et al., 2016). Our stimulation technique could potentially allow perception of the frequency and intensity of such vibrations to be controlled independently as though arising from a fixed stimulus site without smearing the spatial boundaries by recruiting additional afferents with higher currents, as both vibratory and spatial cues play an important role in texture perception (Hollins and Risner, 2000). Moreover, Bhattacharjee et al. (2021) show that vibrotactile perception in humans employ a “temporally local” code that is sensitive to short-lasting features such as individual pulse shapes, and this may allow even finer modulation of perception, as it could be concurrently traded for changes in perceived frequency and intensity.

Such stimulation patterns could also potentially be combined with changing pulse width, which Tan et al. (2014) showed could transform the typical tingling electrical sensation into more natural sensations in upper limb prostheses fitted to amputees, improving their functional ability to control grasp strength and manipulate delicate objects. Our method might help minimise or overcome the resulting biases on perceived frequency or impairment of frequency discrimination sensitivity, which Graczyk et al. (2022) showed could occur when pulse width is varied. Furthermore, Karu et al. (1995) demonstrated that functional electrical stimulation using pulse bursts (which they termed N-lets stimulation, where N represents the number of pulses within the burst) produces less muscle fatigue than traditional singlet pulse patterns, which might be of significance in a prosthesis with both motor and sensory functionality.

Whilst it has been previously shown that perceived frequency within the flutter range (<60 Hz) and vibratory hum range (>60 Hz) can be predicted using the same burst gap code (Ng et al., 2021), a limitation is that we only tested the flutter range in this study. It is speculated that at a high enough frequency, it is possible that the silent gap becomes smaller than the inter-pulse gap and thus results in difficulty in differentiating between separate pulses and bursts. Graczyk et al. (2022) recently demonstrated the possibility of frequency discrimination of electrical pulses breaking down around 50–100 Hz. Additionally, sustained electrical stimulation of the skin has been shown to induce perceptual adaptation that recovers after the stimulus ends (Graczyk et al., 2018). While we included sufficient breaks in the study, the degree of adaptation could increase depending on frequency and amplitude of stimulation (Verrillo and Gescheider, 1977; Hollins et al., 1990). Further research should therefore examine these aspects.

In conclusion, our strategy might enable expansion of the dynamic range for intensity modulation (Sachs et al., 1980; Kaczmarek et al., 1992), which could potentially deliver a sharper spatial contrast between individual stimulation channels when rendering surface texture and object shape reliant on spatial intensity modulation, accompanied by independently controlled tactile frequency perception for mimicking surface interaction during exploratory movements. Accordingly, complex temporal patterns of burst stimulation offer the opportunity for improving information encoding via neural interfaces, which will open new opportunities for control of neuroprostheses in a rapidly developing field (George et al., 2019; Bensmaia et al., 2020; Vickery et al., 2020).

## Data availability statement

The raw data supporting the conclusions of this article will be made available by the authors, without undue reservation.

## Ethics statement

The studies involving humans were approved by the Human Research Ethics Committee, UNSW Sydney. The studies were conducted in accordance with the local legislation and institutional requirements. The participants provided their written informed consent to participate in this study.

## Author contributions

KN, AS, and JF performed the experiments, analysed the data, and prepared the figures. KN drafted the manuscript. KN, IB, and RV conceived and designed the study. All authors contributed to the editing and reviewing of the manuscript and approved the final submitted version.
